# The Medical Schools

**Published:** 1902-09-06

**Authors:** 


### The Medical Schools.

The following account of the medical schools in the United Kingdom will be found useful by those who are about to enter on their studies. So far as the English schools are concerned we give the number of beds in the hospitals to which they are attached, but it is to be noted that mere size is not everything. Of far more importance to the student is the activity of the staff and the willingness of its members to devote time and pains to the work of teaching, and it will be found that the schools attached to some of the small hospitals are admirable as teaching centres. Still, other things being equal, there are certain advantages in having the run of a large hospital, and we are distinctly of opinion that it is an advantage to belong to a fairly large school, one, *i.e.*, having a considerable number of students. At such a place things are apt to be better done, the student is more sure of finding classes ready for him on whatever subject he may be working than where the numbers are smaller, and, what is still more important, the numbers involved renders it possible to "grade" the classes in greater detail.

Under the head "composition fee" we have given the sum charged by each hospital and school for all the classes, lectures, and hospital attendance which are required for the diplomas of the Conjoint Examining Board, but at all the schools special arrangements will be made to suit special requirements.

For further particulars application should be made to the particular schools or hospitals concerning which information is desired, the person to address being usually "The Dean of the Medical School."

At some of the hospitals, although students are accepted for short periods, only full term pupils are eligible for the house appointments. This must be borne in mind and inquired about by those students who, after pursuing the earlier portion of their course at Cambridge or other such place, wish to do their hospital practice in London, for the higher hospital appointments are the great prizes.

### LONDON MEDICAL SCHOOLS.

The medical schools of London now occupy a somewhat different position to that which they held a few years ago in that they are now schools in the Faculty of Medicine of the University of London, and are thus, both in regard to their hospital and teaching staffs, in organic relation with that University.

As time goes on it is possible that some concentration of the earlier and intermediate studies in special University Schools may be effected so that the schools attached to the hospitals may become far more exclusively *medical* than they are at present, their efforts being concentrated upon medicine, surgery, and pathology instead of being scattered among the various anciliary sciences, the study of which forms so large a portion of the medical curriculum. But that time is not yet, and as things stand at present each of the following twelve medical schools in London aims at being complete in itself.

### St. Bartholomew's Hospital Medical School, *West Smithfield, E.C.*

St. Bartholomew's is one of the largest hospitals, and the school attached to it may be regarded as the most important of all the medical schools in London.

The hospital was founded in the year 1123 by Rahere, who subsequently founded the Priory of St. Bartholomew. From the beginning it was a hospital for the sick and not a mere almshouse. This is distinctly expressed in a grant of privileges to it by Edward III. The earliest record of the school dates from 1662. Many additions were made from time to time, and in 1876 the old buildings were removed and replaced on a larger area. This design was completed in 1881 at a cost of £50,000. In 1890 a bacteriological laboratory was added, and in 1891 new laboratories for public health and for biology were built.

A complete convalescent hospital at Swanley, in Kent, forms an important part of St. Bartholomew's Hospital.

The hospital contains 674 beds, in addition to which there are 70 beds for convalescent patients at Swanley.

The appointments connected with the hospital are very numerous and are all free. Many of the senior appointments carry salaries as well as rooms. The school buildings are extensive and very complete, while the scholarships and prizes connected with the school are very valuable.

There is a resident college for students within the precincts of the hospital, and a very admirable recreation ground, ten acres in area, has been acquired at Winchmore Hill, which is easily reached by train.

The composition fee if paid in one sum is 150 guineas, and special arrangements are made for those who have already completed their anatomical and physiological studies.

### Charing Cross Hospital Medical School, *Charing Cross, W.C.*

The position of this hospital and its medical school, situated as they are in the very centre of London and easily accessible from all parts, renders them very attractive in many ways. A great number of street accidents and emergency cases are brought to the hospital and in the out-patients' department a very large number of patients are treated.

The hospital and its convalescent home at Limpsfield together contain 230 beds, and considerable improvements have lately been and still are being made in the buildings. For a hospital of its size there are a considerable number of house appointments and as, from its position, its wards are constantly kept in full swing of work it offers a great and varied field to to the student.

There are five Entrance Scholarships open to students commencing their medical education, three of which (100 guineas, 60 guineas, and 40 guineas) are open to all general students, one is reserved for the sons of medical men, and one is open to dental students only.

There are several Entrance Scholarships, University Scholarships open to students of a certain standing at Oxford and Cambridge, and an Epsom Scholarship which is each winter placed at the disposal of the Royal Benevolent College to be competed for by their foundation scholars who have passed the Preliminary

Scientific M.B. Examination of the University of London. A considerable number of scholarships and prizes are open also to the students of the school during their curriculum.

The composition fee is 110 guineas.

St. George's Hospital Medical School,
*Hyde Park Corner, S.W.*

This hospital occupies an admirable site at Hyde Park Corner, and besides serving the district in its immediate neighbourhood is the principal hospital for that large westward extension of London which has grown up during the past few years, a very considerable proportion of which consists of the homes of working class people. Its position, moreover, is a very attractive one to those who have friends in that part of London, while as a school it stands high. The hospital contains 350 beds, and has a large Convalescent Home of 100 beds near Wimbledon. One ward is set apart for diseases peculiar to women and two are allotted to ophthalmic cases. There are several entrance and other scholarships, one of which is restricted to the sons of medical men. Two scholarships are open only to those who have passed, or completed the curriculum for, the Oxford First M.B. or the Cambridge Second M.B., and another for students of Provincial University Colleges who have completed the first half of their curriculum. The composition fee is £150.

Guy's Hospital Medical School,
*London Bridge, S.E.*

Guy's Hospital contains more than 650 beds, of which 550 are in constant occupation. Thirty-six beds are set apart for diseases of the eye, and 40 for a selection of the most urgent and interesting medical cases which form the subjects of the clinical lectures. This collection of interesting cases in the clinical ward is a matter of great convenience and advantage to the students.

A large ward containing 40 beds is devoted to diseases of women, and a few beds are reserved for cases of difficult labour, an arrangement which offers special advantages to those who are studying midwifery, for which the large outdoor maternity connected with the hospital provides an ample field. A large number of valuable appointments in the hospital are open to deserving students. Not only do the house-physicians, house-surgeons, and resident obstetric assistants receive free board and lodging in the college, but all the dressers are provided with rooms and commons during the period of their "take in."

The college provides accommodation for 60 resident students, and contains a dining hall, reading rooms and a library, and also a gymnasium for the use of the residents and of the members of the Club's Union.

The special departments at Guy's are well developed, and there is a particularly flourishing school of dental surgery.

The Clubs' Union Recreation ground at Honor Oak Park is easily reached from the hospital.

The composition fee is 150 guineas.

King's College Medical Faculty,
*Strand, W.C.*

King's College Hospital is situated in Portugal Street, Lincoln's Inn, W.C., about five minutes' walk from the College. King's and University are two completely equipped colleges, the educational facilities offered by which extend far beyond medicine. Indeed, the Medical is but one of many Faculties. Thus the medical students at both King's College and University College form but a part of a large mass of students who are working at a very considerable range of subjects. Theoretically, one would say that this would be a great advantage, and that the intermingling of those studying arts, sciences, and literature ought to have an important influence upon them by eliminating that narrowness of view which is so apt to characterise those who specialise their studies early in life. We are afraid, however, that at both these colleges the medical students tend very much to keep together and thus to lose one of the great advantages offered to them by these institutions.

King's College Hospital is not very large, the number of beds being only 220, but it is very well arranged for teaching purposes, and a considerable number of appointments are open to the students. At the college also there are a considerable number of scholarships. It may be mentioned that the college is particularly complete in its opportunities for the study of State Medicine.

There is a large recreation ground at Wormwood Scrubbs.

The composition fee is £135 if paid in one sum.

London Hospital Medical School.
*Mile End, E.*

This hospital is situated in the East End of London in the midst of a vast labouring population. There are docks and factories on every side, from which and from the crowded streets there pours in an endless stream of accidents and casualties of all kinds. This is indeed the great accident hospital of London, and from the enormous number of patients who flock to its doors it affords an almost unique field for the study of disease. It is the largest general hospital in the kingdom, containing nearly 800 beds, and during late years constant additions and improvements have been in progress, among the most recent of which are a complete range of operating rooms of the newest type, with accessory wards, sterilising rooms and other conveniences.

All the special departments are very well provided for, and lately the hospital has obtained considerable fame by the publicity given to its arrangements for the special treatment of lupus in accordance with the plan of Professor Finsen, of Copenhagen. A large number of scholarships and prizes are open to the students, and, as one would expect in such a large hospital, the list of appointments is almost endless, in fact, more appointments are open to students here than at any other hospital. No less than 70 qualified appointments are made annually, and more than 150 dressers, clinical clerks, etc., are appointed every three months. All are free to students of the College, and holders of resident appointments have free board. Many of these offices from the opportunities which they offer to their holders for taking personal and responsible charge of patients, are of great value. Some of these appointments are salaried.

The new departments of Bacteriology, Public

Health, Chemistry, and Biology, and the new Pathological Institute at the hospital are now completed and in full use. New students' rooms have recently been added, and a garden and Fives Court opened for the use of the Clubs' Union.

There is also an athletic ground within easy reach, and from the immediate propinquity of the various railway stations it is easy for the students to find lodgings in pleasant suburbs.

The composition fee is 120 guineas, a reduction of 15 guineas being made to sons of medical men.

St. Mary's Hospital Medical School,
*Paddington, W.*

The hospital at the present time contains 281 beds, 128 of which are devoted to medical and 153 to surgical cases. The Clarence Wing, the ground floor of which was opened in 1898, is about to be completed, and when this is done it will increase the number of beds to 362. The out-patient department is very large, and offers an unlimited field for observing all varieties of disease. The situation of the hospital in the midst of a pleasant residential quarter offers great advantages to students who require to live near their work, while the railway, which is only just over the way, renders access to the country and the river peculiarly easy. The number of operations performed has greatly increased during recent years, and the principal operating theatre has been enlarged and entirely reconstructed. All clinical appointments as clerks and dressers are of four months' duration, so that the students have the advantage of a somewhat longer experience of this work than is demanded by the Examining Boards. All the clinical appointments are free, and the residents, who are chosen by competitive examination, receive board and residence in the hospital. There are several good entrance scholarships, and with a view to encouraging students to enter for the higher examinations the special classes held for those so doing are thrown open to all full students without any additional fees.

The composition fee is £140 if paid in one sum, and includes membership of the amalgamated clubs. Special fees are arranged for those students who have completed their examinations in anatomy and physiology at Oxford, Cambridge, or other British Universities.

Middlesex Hospital Medical School,
*Berners Street, W.*

The hospital contains 340 beds. The study of cancer has for long been a special feature at this hospital, and a new wing containing 40 beds and special research laboratories is now entirely devoted to patients suffering from the disease. Thus the Middlesex Hospital offers unrivalled opportunities for the study of this disease both in its clinical and its pathological aspects.

There are several entrance scholarships and 18 resident appointments are open annually to competition among pupils of the hospital. The officers reside in the residential college free of expense. This residential college, which is attached to the hospital and overlooks the hospital grounds, accommodates about thirty students. The non-residential appointments also are numerous, and they are so arranged that each student may, during his course, hold all the out-patient and in-patient clerkships and dresserships.

The composition fee is 135 guineas.

St. Thomas's Hospital Medical School
*Albert Embankment, S.E.*

The present hospital contains in all 602 beds From its foundation down to the year 1862, the hospital occupied the original site where it had stood since the year 1228, when the then new building replaced what was then "an ancient hospital built of old" which had been destroyed by fire 21 years previously. In 1862, however, the site was sold for the extension of London Bridge Station, and in 1871 the new building was opened by her Majesty Queen Victoria. The hospital, as it stands at present, is a modern building on the pavilion plan, occupying a splendid position opposite the Houses of Parliament, and so near to Waterloo Station that junior students can with the greatest convenience live a little way out of town, which is often a great advantage. Considerable alterations have been and are still going on with the object of providing better accommodation in the operation rooms and separate wards for children. One of the children's wards and two of the theatres are already in use.

The school buildings are well fitted for their purpose, and in connection with them are the club premises, reading room, gymnasium, etc., everything being provided to enable the student to spend the whole day within the precincts of the hospital.

An athletic ground, more than nine acres in extent, is provided at Chiswick. The school is well provided with scholarships and prizes, and all the hospital appointments are open to students without charge. The composition fee is 150 guineas.

University College, London, Medical Faculty,
*Gower Street, W.C.*

The hospital contains 188 beds, but the new buildings, one half of which are now completed and in use, will probably increase this number. The new hospital which is being erected by Sir J. Blundell Maple is admirably arranged from the students' point of view, the shortness of the corridors between the wards being productive of much saving of time, and all the laboratory arrangements for the scientific investigation of the cases being most complete and convenient. University College, where the study of the ancillary sciences is undertaken, faces the hospital on the other side of Gower Street. This is an institution of the same type as King's College, although even more complete and wider in its range of teaching. The possibility has been mooted of University College becoming more definitely merged in the University of London than may be the case with the other medical schools of the Metropolis, but so far no alteration in its *locus standi* appears to have been made. There are several entrance and other scholarships, prizes to the annual amount of about £500 being given by the Faculty of Medicine, and all the appointments in the hospital are open to the students without extra fee.

The composition fee is 150 guineas.

Westminster Hospital Medical School,
*Caxton Street, S.W.*

The Westminster Hospital stands just opposite Westminster Abbey, and the school is close by in Caxton Street.

The hospital contains upwards of 200 beds. A medical and a surgical registrar are appointed annually, each with a salary of £40.

Two house-physicians, two house-surgeons, two assistant house-surgeons, and a resident obstetric assistant, are appointed for six months and are provided with rooms and commons in the hospital, except the assistant house-surgeons, who have commons only.

The students' appointments as clerks and dressers are held for four months each.

This is one of the smaller schools, but for its size it is peculiarly well endowed with scholarships, among which is one offering a free medical education to pupils of Epsom College.

The composition fee is 110 guineas, which includes the subscription to the Clubs' Union for five years.

London (Royal Free Hospital) School of Medicine for Women,
30 *Handel Street, Brunswick Square, W.*

The Royal Free Hospital, in connection with which the London School of Medicine for Women is conducted, contains 170 beds, all of which are available for clinical instruction. There are separate departments for the various specialties. Students of the school also attend the practice of one of the Fever Hospitals of the Metropolitan Asylums Board, and receive instruction in lunacy at one of the asylums of the London County Council. Arrangements have also been made for the students of this school to attend the practice of a number of special hospitals.

There are various entrances and other scholarships in connection with the school; the usual dresserships and clerkships at the hospital are open to the students, and after qualification various resident and other appointments are available.

In addition to the ordinary scholarships, various Missionary Societies also offer scholarships on certain conditions, and assist ladies who wish to go to India as medical missionaries.

The school has been recently rebuilt and much enlarged. The laboratories are large, well lighted, and fully equipped. There is a large Library and Common Room, and there are sets of chambers to accommodate seventeen students.

The composition fee is £125.

Cooke's Medical School, London.

This is a school chiefly engaged in the special preparation of students for particular examinations, and is devoted principally to the teaching of anatomy, physiology, and operative surgery on the dead body. Its courses are recognised for Army promotions, and for the Indian Medical Service, and by decision of the various Examining Bodies students rejected at their examinations in anatomy and physiology can get "signed up" from this school for the three months' supplementary work which is required of them before submitting themselves for re-examination. The school is also recognised for the special dissections required for the Fellowship of the Royal College of Surgeons.

### THE PROVINCIAL SCHOOLS.

The medical schools in the provinces occupy a very high position, and from the fact that many of them are directly connected with universities which grant medical degrees, they offer very great advantages to the medical student. We will say at once that except for purely local reasons, such as propinquity to one's home and friends, we can see no advantage in going to any provincial medical school which is not connected with a local university; but in regard to Liverpool, Manchester, and Leeds, the three schools of the Victoria University; Newcastle, which is attached to the University of Durham; and Birmingham, which now has its own university, the advantages are manifold. The admirable hospitals in which the medical instruction is given, the fact that these hospitals are all situated in great manufacturing centres where accidents of all kinds are very common, the advanced position occupied by the leading provincial practitioners and teachers, offer to the student everything which is required for obtaining a thoroughly sound professional education, while those of the provincial schools which are directly connected with local universities give him far greater opportunities than he has in London of obtaining a degree in place of or in addition to a diploma.

The one drawback to a provincial education is the fact that in the eyes of the world London stands first, and that it is to a certain extent a disadvantage to a medical man not to have been brought in touch with the Metropolis of his country. If, however, a provincial student will exercise a little forethought, it is generally easy to arrange that a portion at least of his course shall be passed in London, without in any way interfering with his obtaining his degree. Notably this is the case in regard to Newcastle (where indeed only one whole year need be spent) and Cambridge.

The provincial medical schools must be taken to include the University of Oxford, where the subjects included in the first half of the course are taught, instruction in the more purely medical subjects being sought elsewhere, and the University College School of Medicine at Cardiff where the same conditions hold good. The provincial medical schools at which a complete medical education can be obtained are situated at Cambridge, Newcastle, Liverpool, Manchester, Leeds, Birmingham, Bristol, and Sheffield. Certificates of attendance at any of these schools are accepted by the Royal Colleges, and also by the University of London.

The University of Birmingham (Faculty of Medicine).

To obtain a medical degree in the University of Birmingham it is necessary to have attended the classes of the University for at least the first four years of the curriculum.

The clinical instruction is given at the General Hospital and the Queen's Hospital, under the direction of the Clinical Board. These hospitals have a total of upwards of 450 beds, and are thoroughly well equipped for teaching purposes. The scientific teaching is given by the University professors and lecturers.

The composition fee for the lectures is 80 guineas, and that for the hospital practice 40 guineas.

University College (Faculty of Medicine), Bristol.

It is now arranged that students of the college shall be admitted to the clinical practice of the Bristol Royal Infirmary and the Bristol General Hospital conjointly. These institutions have between them a total of 470 beds, and complete departments for the various specialities, in addition to which they may attend the practice of certain special hospitals in town.

The composition fee for the lectures is 65 guineas, and that for the hospital practice 35 guineas.

The University of Cambridge.

Instruction in clinical medicine, surgery and midwifery is given at Addenbrooke's Hospital so that it is possible to obtain a complete medical education at Cambridge, but the usual practice is for men, after spending three years at the University and passing their examinations in anatomy and physiology, to proceed to London and complete their clinical work in the larger field there open to them.

The advantages of studying at Cambridge are very great, and for those who can afford it there can be very little doubt that there are few, if any, more satisfactory ways of entering the medical profession at the present time than by joining a good college in Cambridge, after passing the Previous, and then at once commencing the study of the sciences which form the groundwork of medicine. After the examinations in anatomy and physiology have been passed it is easy to adjourn to London.

Addenbrooke's Hospital, at which the clinical instruction is given in Cambridge, contains only 158 beds, but it offers quite as large a field as is necessary for the first two or three years, during which the student's time is of necessity almost entirely engaged in working at anatomy, physiology, and the allied sciences.

The fee for pupilship at the Addenbrooke's Hospital is eight guineas.

University College, Cardiff, School of Medicine.

Three out of the five years of the curriculum may be spent at this school, the courses of study being recognised as qualifying for the Preliminary Scientific and Intermediate Examination in Medicine in the University of London, and for the first and second examination under the Conjoint Board.

Clinical instruction is given at the Cardiff Infirmary.

The Yorkshire College (Medical Department), Leeds.

This is one of the colleges of the Victoria University.

The Leeds Infirmary, at which the clinical instruction is given, has accommodation for 482 in-patients including 46 beds at a "semi-convalescent home" in the country which is used to relieve the pressure on the wards. Mental diseases are studied at the West Riding Asylum, and the practice of the Leeds Public Dispensary, the City Fever Hospital and the Hospital for Women and Children is also open to the students. The new buildings for the Medical School were opened only a few years ago, and they are very complete and well equipped.

The composition fee for the college courses is £67 4s., and that for the hospital practice 40 guineas.

University College (Medical Faculty), Liverpool.

This is one of the colleges of the Victoria University. It possesses some very fine and well-equipped laboratories. Many very liberal gifts have been made to this institution, and everything is being carried out in a most complete manner, so that it has become a thoroughly organised and well-equipped medical school.

The Royal Infirmary, which adjoins the School, contains 300 beds, with 40 special beds for the treatment of diseases of women. The Lying-in, Eye and Ear, Children's, and other special hospitals are in the immediate vicinity, and their practice is open to students.

Numerous scholarships and prizes, amounting to over £600, are awarded annually.

In connection with this college is the now well-known Liverpool School of Tropical Medicine.

The composition fee for the college classes is £70, and that for the hospital practice is £42.

Owens College (Medical Department), Manchester.

This is one of the colleges of the Victoria University.

The Manchester Royal Infirmary, at which the medical instruction is given, contains 292 beds, and in connection with it there is a convalescent hospital (the Barnes Convalescent Hospital), containing 136 beds. Instruction in mental diseases is given at the Royal Lunatic Asylum, and in infectious diseases at the Fever Hospital.

Owens College, Manchester, of which the medical school is a department, is a large and most complete educational institution. Extensive additions have lately been made to the buildings, so as to give the fullest possible facilities for teaching in those departments which are more especially important to the students entering for medicine, namely, anatomy, physiology, pathology, and materia medica. Indeed in every direction Owens College is most thoroughly equipped for its work.

Three halls are licensed by the college for the residence of students, namely, Dalton Hall, Victoria Park, and Hulme Hall, Plymouth Grove, for men, and Ashbourne House, Victoria Park, for women students.

The composition fee for the college courses is £70, and for the hospital practice £42.

University of Durham College of Medicine, Newcastle-upon-Tyne.

The Royal Infirmary, where the clinical instruction is given, contains 280 beds, but a large new hospital is being erected.

Residence at Newcastle for one year out of the five is necessary for all medical students desiring to graduate at the Durham University.

The composition fee for the college courses is

70 guineas, and that for the hospital practice 25 guineas, with small additional payments in certain cases.

### The University of Oxford Medical School.

At the Radcliffe Infirmary instruction is given in connection with the University Medical School. The whole course of study, at the museum and infirmary, is intended for students until they have passed the Second Conjoint Examination or the First Oxford M.B. The Radcliffe Infirmary contains 147 beds.

### University College (Department of Medicine), Sheffield.

The hospitals at which the clinical instruction is given are the Sheffield Royal Infirmary, containing 240 beds, the Sheffield Royal Hospital with 125 beds, the Jessop Hospital for Women, and several special hospitals. In connection with the College there is now a complete Dental Department where students are able to go through their full curriculum.

The composition fee for the lectures is £63, and for hospital practice £45.

### SCOTTISH MEDICAL SCHOOLS.

### University of Edinburgh.

Most of the classes are held at the University and in the various laboratories connected with it. The clinical instruction is given in the Royal Infirmary, where there are 780 beds ; in the Royal Hospital for Sick Children, with 120 beds ; the Maternity Hospital, the City Fever Hospital, and the Morningside Asylum.

### School of Medicine of the Royal Colleges, Edinburgh.

This school of medicine, sometimes spoken of as the extra-mural school, is composed of lecturers licensed by the Royal Colleges of Physicians and Surgeons, and is carried on in several buildings in the neighbourhood of the Royal Infirmary.

The lectures thus given are recognised by the various Royal Colleges in Edinburgh, London, and Dublin, and in accordance with the statutes of the University of Edinburgh one half of the qualifying classes required for graduation may be attended in this school. The lectures given here are also recognised by the University of London. Clinical instruction is given to students of the school, as to those of the University, in the Royal Infirmary and other clinical hospitals in Edinburgh. The whole arrangement is curious and special to Edinburgh, and there is reason for believing that much of the pre-eminence of Edinburgh as a teaching centre has hinged upon the constant competition between the lecturers within and without.

### Medical College for Women, Edinburgh.

The clinical instruction is given in the wards of the Royal Infirmary and other institutions.

### University of Glasgow.

The University is very near to the Western Infirmary, with 400 beds, where clinical instruction is given, as also at the Lunatic Asylum and various special hospitals.

### Queen Margaret College, Glasgow (the Women's Department of the University).

This College provides a separate building, with class rooms, library, and other teaching appliances, for women students, who are there taught apart from male students. But the women have all the rights and privileges of university students, and do their clinical work in the Royal Infirmary, where special wards are reserved for their use, and in other institutions in the city.

### St. Mungo's College, Glasgow.

The buildings of this College are situated within the grounds of the Royal Infirmary, the wards of which are available for clinical instruction, which, however, is supplemented by attendance at other institutions.

### Anderson's Medical College, Glasgow.

The buildings of this College are situated close to the Western Infirmary, and very near the University. The clinical instruction may be obtained at the Western Infirmary and other institutions as in the case of the University students.

### University of Aberdeen.

Clinical instruction is provided at the Royal Infirmary, which has 240 beds, the Royal Lunatic Asylum, the Sick Children's Hospital, and other institutions.

### University of St. Andrews (United College, St. Andrews, and University College, Dundee).

By combination between the ancient University of St. Andrews, the medical department of the University College of Dundee, and the Dundee Royal Infirmary, a complete medical school has been arranged. At least two of the five years of medical study must be spent either at the United College, St. Andrews, or at University College, Dundee, both of which are colleges of the University, and the rest of the time may, if desired, be passed at other medical schools, but the whole course may be obtained in direct connection with the University of St. Andrews, which in either case will grant its degrees to successful students.

### IRISH MEDICAL SCHOOLS.

The School of Physic in Ireland, Dublin.

The Catholic University Medical School, Dublin.

The Schools of Surgery of the Royal Colleges of Surgeons in Ireland, Dublin.

Queen's College, Belfast.

Queen's College, Cork.

Queen's College, Galway.

### MEDICAL SCHOOLS AND MEDICAL QUALIFICATIONS FOR WOMEN.

The following are the qualifying bodies which admit women as candidates for their degrees or diplomas, viz., all the Universities of England with the exception of the Universities of Oxford and Cambridge; the Universities of Scotland; the Royal Universities of Ireland; the Society of Apothecaries, London; and the Conjoint Board of the Royal Colleges of Physicians and Surgeons of Scotland and Ireland.

The regulations of the General Medical Council in regard to medical education and examinations, apply to women exactly as they do to men.

In regard to place of study women medical students can obtain a complete medical education by becoming students of the London School of Medicine for Women, which is connected with the Royal Free Hospital; the Medical College for Women, Edinburgh; or Queen Margaret College, Glasgow (which is now an integral part of the University of Glasgow), all of which are open to women only; or of the Schools of Surgery of the Royal College of Surgeons in Ireland, where special rooms are set apart for lady students; or of the Queen's Colleges of Belfast, Cork, and Galway. They can also enter at the Universities of Edinburgh, Aberdeen, and St. Andrews; at the University of Birmingham; at some of the colleges of the Victoria University, and at the University of Durham College of Medicine and College of Science which are situated in Newcastle-upon-Tyne. The medical classes of the University of St. Andrews are open to women on an equal footing with men, and it may be arranged either to take the first two years at United College, St. Andrews, and the rest at Dundee, or the whole five years may be spent at University College, Dundee. At the Dundee Royal Infirmary and the Dundee Royal Lunatic Asylum, women students are received. For English women students who feel equal to the examinations of the London University, the London School of Medicine for Women will be found thoroughly suitable, or they might spend a portion of their time at Newcastle after matriculating at the Durham University and then finish in London. The secretary of the School of Medicine for Women, 30 Handel Street, London, W.C., will give every information.

